# Investigation of safety for electrochemotherapy and irreversible electroporation ablation therapies in patients with cardiac pacemakers

**DOI:** 10.1186/s12938-020-00827-7

**Published:** 2020-11-16

**Authors:** Tomaz Jarm, Tadej Krmac, Ratko Magjarevic, Bor Kos, Helena Cindric, Damijan Miklavcic

**Affiliations:** 1 University of Ljubljana, Faculty of Electrical Engineering, Trzaska 25, 1000 Ljubljana, Slovenia; 2 University of Zagreb, Faculty of Electrical Engineering and Computing, Unska 3, 10000 Zagreb, Croatia

**Keywords:** Electrochemotherapy of tumors, Irreversible electroporation, Non-thermal ablation therapy, Cardiac pacemaker, Implantable devices, Safety, Numerical modeling, Experimental work

## Abstract

**Background:**

The effectiveness of electrochemotherapy of tumors (ECT) and of irreversible electroporation ablation (IRE) depends on different mechanisms and delivery protocols. Both therapies exploit the phenomenon of electroporation of the cell membrane achieved by the exposure of the cells to a series of high-voltage electric pulses. Electroporation can be fine-tuned to be either reversible or irreversible, causing the cells to either survive the exposure (in ECT) or not (in IRE), respectively. For treatment of tissues located close to the heart (e.g., in the liver), the safety of electroporation-based therapies is ensured by synchronizing the electric pulses with the electrocardiogram. However, the use of ECT and IRE remains contraindicated for patients with implanted cardiac pacemakers if the treated tissues are located close to the heart or the pacemaker. In this study, two questions are addressed: can the electroporation pulses interfere with the pacemaker; and, can the metallic housing of the pacemaker modify the distribution of electric field in the tissue sufficiently to affect the effectiveness and safety of the therapy?

**Results:**

The electroporation pulses induced significant changes in the pacemaker ventricular pacing pulse only for the electroporation pulses delivered during the pacing pulse itself. No residual effects were observed on the pacing pulses following the electroporation pulses for all tested experimental conditions. The results of numerical modeling indicate that the presence of metal-encased pacemaker in immediate vicinity of the treatment zone should not impair the intended effectiveness of ECT or IRE even when the casing is in direct contact with one of the active electrodes. Nevertheless, the contact between the casing and the active electrode should be avoided due to significant tissue heating at the site of the other active electrode for the IRE protocol and may cause the pulse generator to fail to deliver the pulses due to excessive current draw.

**Conclusions:**

The observed effects of electroporation pulses delivered in close vicinity of the pacemaker or its electrodes do not indicate adverse consequences for either the function of the pacemaker or the treatment outcome. These findings should contribute to making electroporation-based treatments accessible also to patients with implanted cardiac pacemakers.

## Background

### Electroporation

When biological cells are exposed to electric pulses with intensity above certain threshold, the induced transmembrane voltage leads to electroporation—a physical phenomenon of increased cell membrane permeability for otherwise poorly permeant molecules [1, 2]. If the intensity of the external electric field is below another threshold this effect is transient, the membrane reseals and the cell survives (the reversible electroporation), otherwise the electroporation leads to the cell death (the irreversible electroporation—IRE). Both types of electroporation are exploited in different areas as diverse as biomedicine and food processing technologies [1, 3]. Two clinical applications are considered in this paper—electrochemotherapy (ECT) and IRE ablation of tumors. In both, the effects of the therapy are localized to the area of electroporation with minimal side effects to the surrounding tissue.

### Electrochemotherapy of tumors

In electrochemotherapy (ECT) of tumors reversible electroporation is combined with chemotherapy. A single dose of the chemotherapeutic drug (bleomycin or cisplatin) is injected intravenously or intratumorally before the application of electroporation pulses [4]. Transient increase of membrane permeability of tumor cells facilitates an increased cellular uptake of the hydrophilic drug molecules from the extracellular space [1, 5]. This leads to significantly potentiated cytotoxic effects due to entrapment of the drug after resealing of the membrane [6]. This is the main mechanism of antitumor effectiveness of ECT. However, two additional contributing mechanisms were identified—the vascular effects and the involvement of the immune response [6, 7]. Routine use of ECT and the number of clinical trials for new indications is constantly growing. ECT is an efficient and safe therapy for treatment of different types of solid malignancies in various superficial and internal tissues, including tumors in skin, head and neck, brain, bone and internal organs (visceral and deep-seated tumors) in human and veterinary medicine [8]. In a recent randomized Phase 3 study of electrochemotherapy on basal cell carcinoma, it was shown that ECT is equally efficient as surgery [9]. A typical protocol for ECT involves the application of a sequence of pulses for each active pair of electrodes (8 pulses per pair). Pulses of short duration (100 µs) and high voltage (e.g., at 1000 V/cm voltage-to-distance ratio) are used and delivered either individually at 1 Hz or in sequences of 4 pulses with a 5-kHz repetition rate within a sequence [10]. Pulse delivery is synchronized with the ECG when necessary. In clinical settings and for the largest inter-electrode distances (2–3 cm), the maximum voltages and currents can be as high as 3000 V and 50 A, respectively [8], and there can be as many as 12 electrode pairs that the electroporation pulses are delivered to. Various types of needle-type electrodes were developed for different applications [6, 8, 11].

### IRE ablation therapy

If the intensity, number and/or duration of applied pulses exceed irreversible threshold values, the affected cells die due to electroporation (IRE), largely due to irrecoverable loss of homeostasis [2]. IRE is thus used as a non-thermal ablation therapy with important advantages over the well-established conventional ablation methods [12, 13]. In IRE ablation, the cell death is predominantly a result of electroporation and not the temperature increase. However, local heating of tissue does occur, especially in the immediate vicinity of the electrodes and when large numbers of pulses are used [12, 14, 15]. IRE ablation has been used in clinical trials for treatment of tumors in internal organs such as liver, pancreas, kidneys and prostate [11, 16–19]. Compared to ECT, the number of pulses and voltages in IRE ablation of tumors are significantly larger. Typically, at least 70–90 pulses of 70–100 µs duration are used at the voltage-to-distance ratio of 1500 V/cm. The spacing between needle electrodes is 1.5–2 cm and their active length is 1–1.5 cm [12].

ECT and IRE ablation of tumors can benefit from the individualized treatment planning based on mathematical modeling and from coupling the treatment plan with a navigational system to provide more accurate positioning of the electrodes and optimized electrical parameters for the treatment [20, 21]. In this way, optimal coverage of the target tissue with sufficiently high electric field strength for the desired effect and minimal damage in the normal tissue can be achieved. Treatment planning also helps to avoid excessive heating and thermal damage of critical structures/tissues and to keep the current below the maximum output level of the device.

### Cardiac and other safety considerations for electroporation-based therapies

When treating deep-seated internal tumors in vicinity of the heart (e.g., in the liver), there is an increased possibility for interactions of electroporation pulses with the cardiac activity. The risk of harmful interferences is minimized by synchronization of the delivery of electroporation pulses with the electrocardiogram (ECG). Synchronization is recommended for all electroporation-based therapies in thoracic cavity or close to the heart [12, 22]. However, as a safety precaution, the use of electroporation-based therapies remains contraindicated for patients with implanted cardiac pacemakers if the treatment zone is close to the implanted device [23]. Both manufacturers of clinically approved devices for generation of electroporation pulses, namely IGEA (Carpi, MO, Italy) for the Cliniporator system (for ECT) and Angiodynamics (Latham, NY, USA) for the NanoKnife system (for IRE ablation) consider cardiac pacemakers as contraindication for treatment [24, 25]. Angiodynamics further expands this to any implanted devices with metallic parts.

The presence of metallic implants within or in vicinity of the treatment zone may indeed negatively affect the outcome of electroporation-based treatments [26–28]. Metallic casing of the pacemaker has higher electrical conductivity than surrounding tissue and may therefore change the electric field distribution, which could potentially lead to undertreatment of the target tissue. It may also present an increased risk for thermal damage in the surrounding tissue, especially for IRE ablation. IRE ablation is usually considered a non-thermal method. However, several experimental in vivo and in silico studies have shown a significant increase in temperature during treatment [14, 15, 20, 29, 30]. Thermal coagulation has been observed a few millimeters from the electrodes in animal experimental studies [29–31]. The extent of thermal damage depends on tissue type, pulse parameters, electrode exposure length and inter-electrode distance. Furthermore, the presence of metal has been found to increase generation of heat [29].

### Aims of the study

In this preliminary study two questions related to the contraindication of electroporation-based therapies for patients with pacemakers were addressed:

- Do the electroporation pulses electrically interfere with the function of the pacemaker in a way that could lead to its malfunction or even damage?
- Is the distribution of electric field in the tissue modified by the metal housing of the pacemaker to the extent that either the effective treatment zone is modified and/or that the tissue may be exposed to excessive heating?

The first question was addressed empirically by exposing a functioning pacemaker with its ventricular lead to electroporation pulses under various conditions and observing the effects, and the second question by numerical modeling of physical conditions encountered during application of electroporation pulses near the implanted pacemaker and pacemaker leads.

## Results

### Experimental evaluation of the effect of electroporation pulses on the pacemaker

**Fig. 1:**
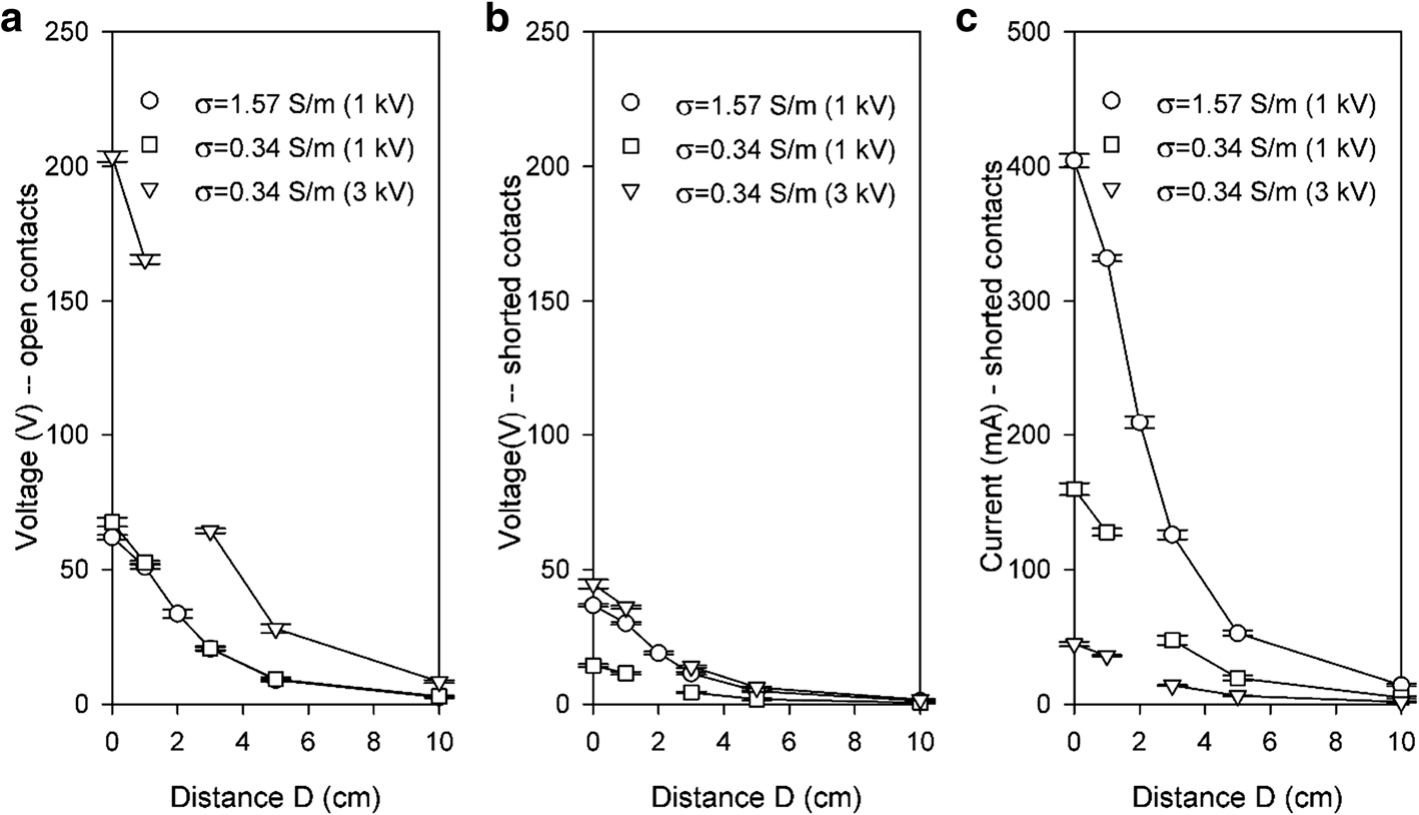
Voltages and currents induced by electroporation pulses on the disconnected ventricular lead for open contacts (**a**—voltage only) and shorted contacts (**b**, **c**) as a function of the distance between the electroporation and the pacemaker electrodes. For the amplitudes of electroporation pulses 1000 V (both conductivities) and 3000 V (lower conductivity only). Mean average values and ± SD bars are shown (*N* = 6–8). See Fig. 5 for the definition of distance *D*

Figure 1 presents steady-state voltages and currents (the interference) measured at the pacemaker ventricular electrodes without the pacemaker during application of a single 100-µs-long electroporation pulse. Two extremes are presented: with open ventricular lead contacts (the maximum voltage and zero current) and with shorted contacts (the maximum current at reduced voltage that represents the voltage drop on combined resistances of the anodic and cathodic leads). With the pacemaker connected, the combined resistance includes the internal resistance of the device, therefore voltages between those in Fig. 1a, b and currents lower than those in Fig. 1c are anticipated. As expected, the amplitude of the interference decreased rapidly with the distance of the electroporation electrodes from the pacemaker’s ventricular lead.

**Fig. 2:**
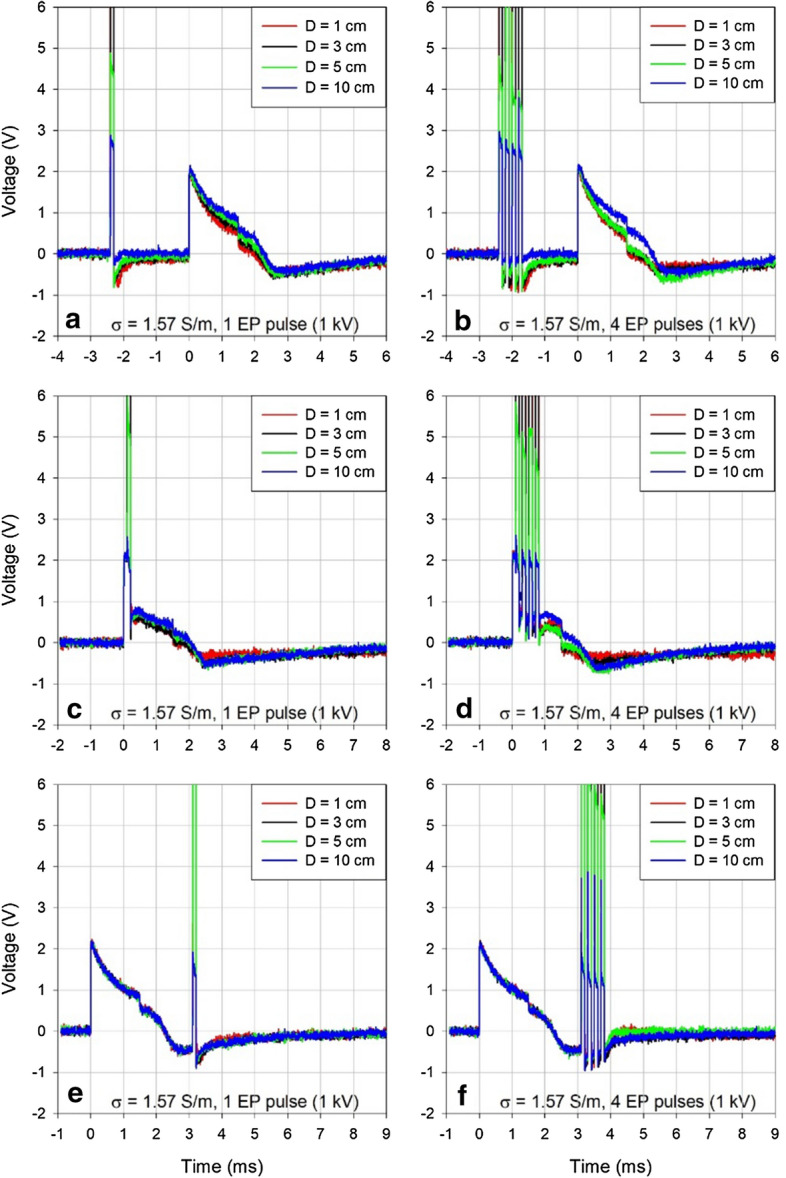
The interference of a single electroporation pulse (**a**, **c**, **e**) and a sequence of four electroporation pulses (**b**, **d**, **f**) with 1000 V amplitude, 100 μs duration, and 5 kHz repetition rate. Electroporation pulses were delivered before (**a**, **b**), during (**c**, **d**) or after (**e**, **f**) the ventricular pacing pulse in the medium with conductivity of 1.57 S/m. For comparison with the effects observed in the media of lower conductivity at 1000 V and 3000 V amplitudes see Additional file 1: Figures S1 and S2

Figure 2 presents a typical example of the effect of electroporation pulses on ventricular pacing pulse for one or four electroporation pulses at 1000 V amplitude delivered in the medium of the higher conductivity (physiological saline with the conductivity of 1.57 S/m).

**Fig. 3:**
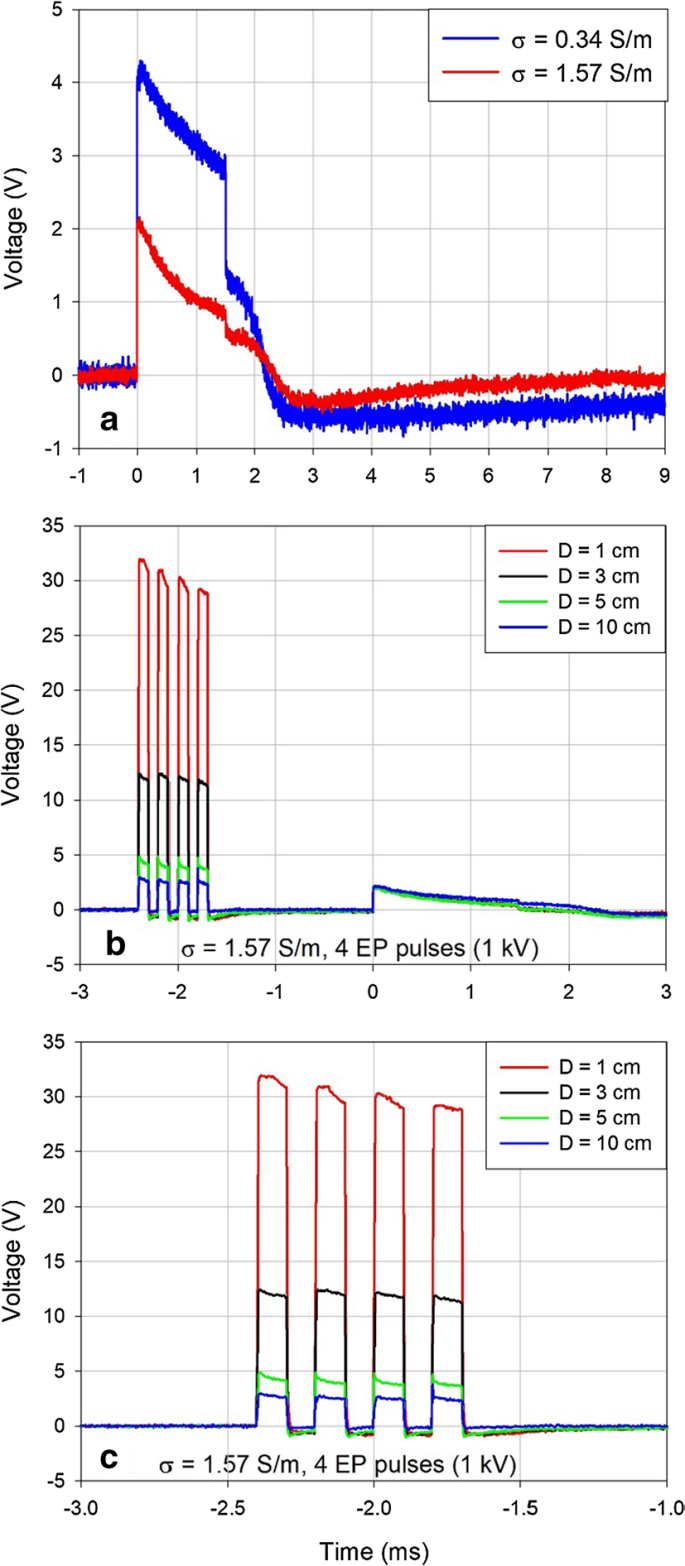
**a** Unperturbed ventricular pacing pulse in two media of different conductivities. **b** Comparison of relative amplitudes of the electroporation pulse artifacts and the ventricular pacing pulse (same data as shown in Fig. 2b). **c** Electroporation pulse artifacts from panel **b** shown in more detail

Figure 3 shows unperturbed ventricular pacing pulses measured in both conductive media used in the study (panel a), a zoomed-out version of Fig. 2b for comparison of relative amplitudes of the ventricular pacing pulse and the artifacts caused by electroporation pulses (panel b), and a more detailed view of only the said artifacts (panel c). Note the declining amplitude of the artifacts caused by the technical limitation of the Cliniporator pulse generator, which does not recharge its output capacitors during delivery of pulses in high-frequency sequences.

Electroporation pulses resulted in distance-dependent voltage artifacts of expected polarities, duration and reproducible amplitudes (similar to those reported in Fig. 1b). No drastic changes in the characteristics of the pacing pulses were induced by the electroporation pulses delivered before or after the stimulating phase of the pacing pulse (Fig. 2 panels a, b, e, f).

Additional file 1: Figures S1 and S2 present the same kind of information as Fig. 2 for electroporation pulses delivered in the medium with decreased conductivity of 0.34 S/m, which is comparable to conductivities encountered in clinical practice in tissues such as human liver. This reduced conductivity also enabled the use of the highest available voltage for electroporation pulses, which is 3000 V (again comparable to clinical situation). It can be seen that the same kind of effects as described in Fig. 2 were also present in the medium of lower conductivity, but the effects were more pronounced here and the higher voltage resulted in considerably larger artifacts. In general, all observed effects were distance-dependent (decreasing the distance *D* between the electroporation and the pacemaker electrodes resulted in progressively larger interferences).

The shape of the pacing pulse was significantly perturbed and its amplitude reduced only for electroporation pulses delivered during the stimulating phase of the pacing pulse (panels c and d in Fig. 2, Additional file 1: Figures S1, S2). The effects were similar for all investigated conditions except that they were less or more pronounced, depending on the number of pulses or the amplitude of electroporation pulses. However, the most relevant observation was that all the described effects appeared only for pacing pulses coinciding with the electroporation pulse(s). There was no residual effect on the following pacing pulses. The pacemaker appeared completely immune to electroporation pulses.

### Numerical modeling

**Fig. 4:**
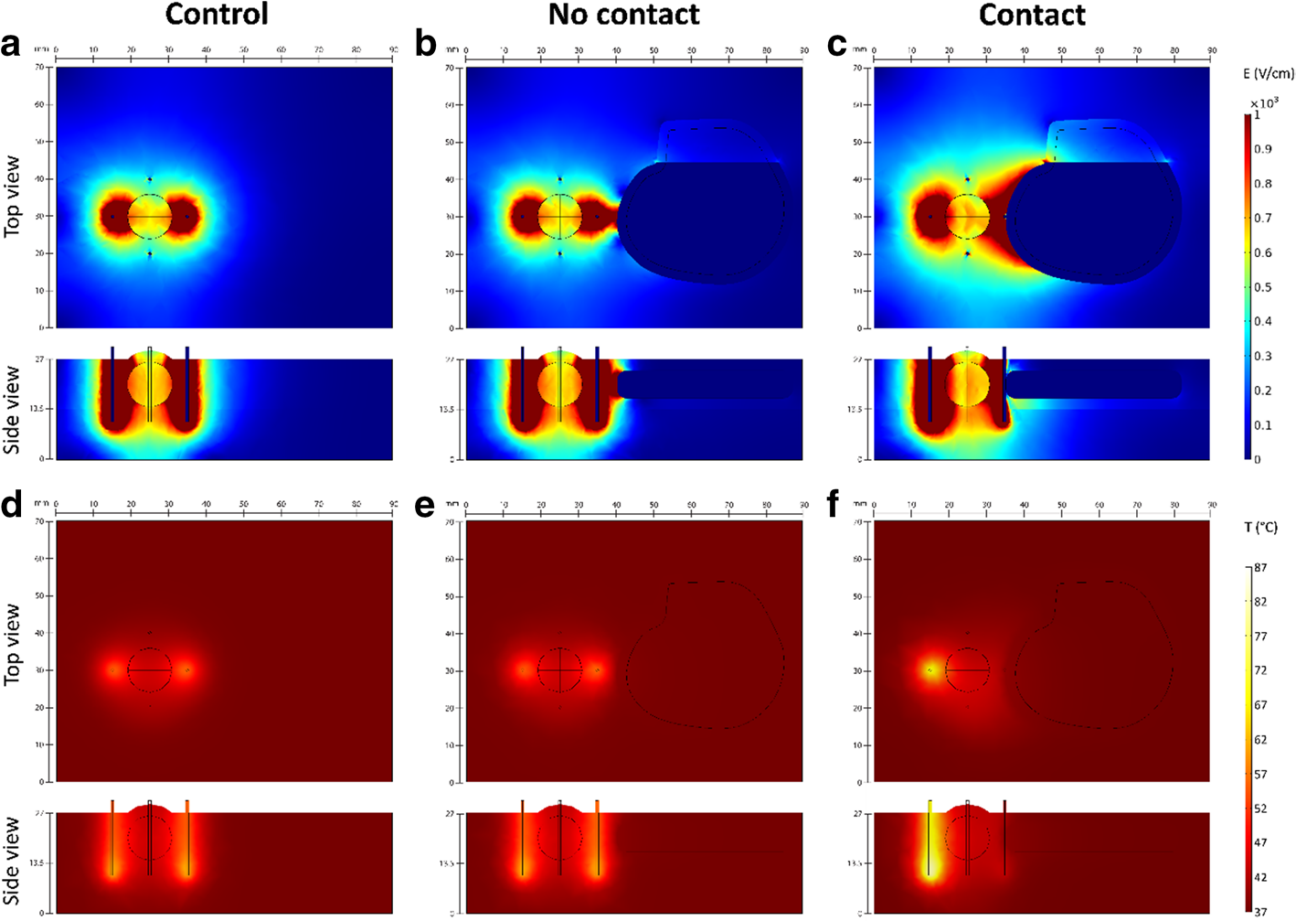
The impact of the presence of metallic-encased pacemaker during IRE ablation of tumor. Images show computed results at the end of pulse delivery for one of six active electrode pairs in the simulated procedure. The simulated pulse protocol was 90 × 90 μs pulses delivered at 1 Hz with a pause of 3 s after each set of 10 pulses. Applied voltage to distance ratio was 1500 V/cm. **a**–**c** Electric field distribution in target tissue; **d**–**f** Temperature in the target tissue. In the control model, the pacemaker is not present in the simulation. In the no-contact model pacemaker is positioned 5 mm from the rightmost electrode. In the contact model, the pacemaker is in conductive contact with the rightmost electrode. The top and side views correspond with the model geometry shown in Fig. 6b

The influence of the presence of pacemaker is similar for both treatments (ECT and IRE). In Fig. 4 only the results for IRE are shown because the impact of the pacemaker is more noticeable in IRE protocols due to higher number of pulses used.

In both treatments, the presence of the pacemaker without contact with the electrodes does not significantly affect the delivered electric currents when compared to control values (0–2% change). Changes in electric field distribution are mainly observed in the healthy tissue, while electric field in the tumor remains mostly unaffected (Fig. 4b). Complete coverage of the tumor with sufficiently high electric field is achieved for both treatments. When compared to the control model (without pacemaker), the presence of the pacemaker does not cause any additional heating of tissue (Fig. 4e). Although both treatments are considered non-thermal, non-negligible tissue heating is observed at the electrodes, especially in IRE. The maximum calculated increase from base tissue temperature (37 °C) is 9.6 °C and 40 °C for ECT and IRE ablation, respectively; the temperature rise is limited to immediate vicinity of the electrode tips.

When the pacemaker is in direct contact with one of the electrodes, its influence is more prominent. A significantly increased current draw is observed in the electrode pairs containing the contact electrode—the calculated electric current is approximately 50% higher when compared to control values. Electric field distribution changes drastically in electrode pairs containing the contact electrode as well. Since the pacemaker itself acts like an electrode, higher electric field is produced in the tumor and healthy tissue (Fig. 4c). A large volume of tissue surrounding the contact point is subject to IRE. Electric field in the tumor in this specific case is not impacted negatively: due to overall higher electric field, complete coverage of the tumor is achieved with fewer electrode pairs compared to the control and the no-contact models. Higher temperatures are observed in tissue for both treatments when compared to the control models. In the ECT model, up to 5.2 °C higher temperatures are observed in a pair-to-pair comparison; the temperature rise is most significant in the pairs containing the contact electrode (average 3.2 °C increase). However, this rise in temperature is limited to the immediate vicinity of the electrodes. The overall maximum calculated temperature is the same as in the control model without pacemaker. In the IRE model, contact with one of the electrodes results in significant heating around the opposite electrode of the pair, however no significant heating is observed at the contact point (Fig. 4f). Up to 31.4 °C higher temperatures are observed in a pair-to-pair comparison to the control model. The temperature rise is most significant in pairs containing the contact electrode (average 24.1 °C increase). The overall maximum calculated increase from base tissue temperature is 70.4 °C (compared to 40 °C in the control model).

In the model some areas in immediate vicinity of the electrodes are heated to temperatures of more than 100 °C, because there is no term for boiling included in the numerical model. In reality, vaporization would occur at these high temperatures, which would drastically decrease bulk conductivity and further current increase.

The numerical results are shown in more detail in the tables in Additional file 2.

## Discussion

### Experimental evaluation of the effect of electroporation pulses on the pacemaker

The application of electroporation pulses induces significant changes in the shape of the pacemaker ventricular pacing pulse in experimental conditions only when the electroporation pulses coincides with the stimulating phase of the pacing pulse with no residual effects in the following pacing pulses for all tested conditions.

**Fig. 5:**
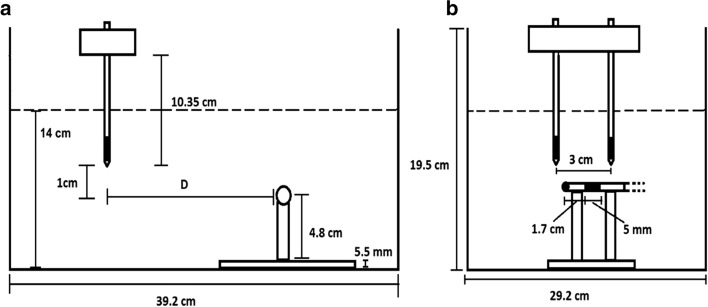
Relative positions of the electroporation electrodes (suspended vertically from above) and the stimulating distal end of the pacemaker bipolar ventricular lead (fixed horizontally) in a container filled with conductive solution to the height of 14 cm [38]. Side view (**a**) and frontal view (**b**) of the container is shown. The active parts of the electrodes are shaded black. The distance *D* was adjusted between 0 and 10 cm as shown in Figs. 1 and 2

A decaying baseline voltage shift of the opposite polarity was observed when the pulses were delivered before the pacemaker pulse. Since the shift did not dissipate before the ventricular pacing pulse was delivered by the pacemaker, it visibly affected the absolute amplitude of the following pacing pulse (Fig. 2a, b). As all other effects, the magnitude of this shift decreased with the decreasing distance *D* (the horizontal distance between the electroporation electrodes and the pacemaker ventricular lead electrodes, see Fig. 5). We cannot fully explain this shift, but it was probably partially due to the combined resistive and inductive properties of the anodic and the cathodic parts of the ventricular lead. A similar effect of a decaying negative voltage shift that lasted more than 2 ms following the electroporation pulse was observed in the artifact induced by the electroporation pulse when the pacemaker was disconnected. In that case the ventricular lead was shorted at the other end thus permitting the maximum current to flow through serially connected anodic and cathodic leads during the delivery of the electroporation pulse. This effect was much less pronounced when the current was not flowing in the ventricular lead (the contacts of the ventricular lead left open). See also Additional file 1: Figure S3.

In general, all observed effects were distance-dependent (decreasing the distance *D* between the electroporation and the pacemaker electrodes resulted in progressively larger interferences). However, it needs to be pointed out that sometimes we observed some deviation to this rule. Most notably this can be seen in Additional file 1: Figure S1A, B (1000 V pulses) where the effects observed for the inter-electrode distance *D* = 3 cm did not fit with the distance-dependency rule and we do not have an explanation for this.

After testing in harsh conditions with *D* = 1 cm at the maximum available electroporation voltage of 3000 V and in a medium with conductivity comparable to tissue conductivity in ECT/IRE of deep-seated tumors we found no evidence of any malfunction of the pacemaker. However, without the proprietary data about the electrical protection of the Adapta pacemaker we cannot claim that the maximum voltages observed on the ventricular lead represent absolutely no risk for the pacemaker. Nevertheless, further mitigating circumstances should be considered for realistic applications. In clinical situations, the electrodes for delivery of electroporation pulses would never be placed as close to the pacemaker electrodes as in our study. Distances of at least about 3 cm or more between the two sets of electrodes can be expected unless the treatment zone was in the heart itself. At *D* = 3 cm the amplitude of the observed interference was less than 30 V. Furthermore, in clinical ECT (and also for IRE) of deep-seated tumors in thoracic cavity the delivery of electroporation pulses must always be synchronized with the absolute refractory period of the ventricles, which follows the depolarization of the atria and the ventricles. Therefore, for correct synchronization the electroporation pulses would be delivered after the unperturbed ventricular pacing pulse (or normal ventricular R wave) and therefore could not affect the shape of pacing pulses, atrial or ventricular. Furthermore, the moment of correctly delivered electroporation pulses immediately after depolarization of the ventricles would coincide with the blanking periods for both the atrial and ventricular channel (e.g., in case of the DDD pacing mode) and should therefore not interfere with the programmed function of the pacemaker [32]. Finally, pacemakers must be able to withstand the external defibrillation treatment. Even though not directly comparable, the total energy delivered to a pair of electroporation electrodes in ECT is typically only a fraction of the energy of a single defibrillation pulse. All this suggests that the application of ECT for deep-seated tumors in close vicinity of the heart should probably not be contraindicated, as also stated in the updated standard operating procedures for ECT of cutaneous tumors [10]. This conclusion could be extended to the IRE ablation therapy. Namely, even though the total number of delivered electroporation pulses (and consequently the total energy) is significantly larger than in ECT (typically 90 vs 8 pulses per each electrode pair for IRE vs ECT, respectively), the pulses are delivered one at a time for IRE ablation and they are individually synchronized with the ECG [10, 12]. Therefore, the delivered energy in case of IRE is spread over a much longer period (more than an order of magnitude) than in case of ECT and therefore represents a less intensive instantaneous stress for the pacemaker.

### Numerical modeling

Numerical results suggest for both therapies (ECT and IRE) that the presence of metal-encased pacemaker does not affect the coverage of the tumor tissue regardless of contact with the electrode and should thus not impair the effectiveness of the electroporation-based treatment. However, if the pacemaker is in contact with one of the electrodes, the entire housing acts as a large electrode resulting in an increased current draw from the electroporator. Consequently, overall higher electric fields are produced in target tissue, potentially achieving better coverage than without the pacemaker. However, this increased current draw also increases the probability of interruption of pulse delivery due to exceeded hardware-limited maximum values [33].

In both treatments, tissue heating is not increased in the presence of the pacemaker without contact with one of the electrodes. If contact with one of the electrodes is established, however, higher temperatures are observed in tissue. In ECT the rise in temperature is not as pronounced, which indicates that treatment safety should not be affected. In IRE a significant rise in temperature is observed at the site of the second active electrode in the pair. This observation agrees with the observation of heating around the electrodes when a metal stent was present within the treatment zone [26]. The metallic casing itself does not heat up during treatment, but rather acts like a heat sink, therefore thermal damage due to heating of metallic casing is unlikely.

Due to its limitations, our study should be considered preliminary and thus conclusions need further confirmation. One of the limitations of the numerical part of the study is the lack of validation of the model. Although the same model has been used in previous studies, and has also been validated for various tissues, such as liver, muscle and kidney [20, 34–37], it has not yet been validated for this specific tissue setting. Moreover, the potential negative impact of the presence of a metal-encased pacemaker on the efficacy of electroporation-based treatment has only been investigated in one simplified geometry. A patient-specific treatment plan is in any case advised.

 The situation with the pacemaker in contact with more than one electrode has not been evaluated in this study. However, it is worth noting that such a condition would result in short circuit conditions. Pulse delivery would be terminated for all shorted electrode pairs due to excessive current, which would result in undertreatment of the target tissue.

## Conclusions

In our study we found no evidence of harmful effects of electroporation pulses, such as those used in ECT or IRE of tumors, on functioning of a pacemaker even for pulses applied in immediate vicinity of the pacemaker electrodes. Transient voltage artifacts of up to almost 200 V were observed on the pacemaker electrodes during delivery of electroporation pulses in the most extreme situation (the maximum pulse voltage of 3000 V and the unrealistically small distance between the electroporation and the pacemaker electrodes of 1 cm). In conditions resembling those encountered in clinical practice for the smallest realistic distance between the treatment and the pacemaker electrodes (i.e., 3 cm) the amplitude of voltage artifacts did not exceed 30 V. Due to similarity of electroporation pulses used for ECT and IRE treatments this observation is equally relevant for use of IRE ablation in patients with pacemakers. Numerical computation showed elevated temperatures in immediate vicinity of the electrode tips also without the presence of the pacemaker—up to 9.6 °C and 40 °C increase from base tissue temperature for ECT and IRE, respectively. The presence of the pacemaker without contact with the electrodes did not further contribute to tissue heating. When the pacemaker was in direct contact with one of the electrodes up to 9.6 °C and 70.4 °C increase from base tissue temperature was observed in ECT and IRE, respectively. In the modeled geometries, the presence of a metal-encased pacemaker did not negatively affect tumor coverage regardless of contact of one electrode with the pacemaker housing.

Our study should be considered preliminary and thus conclusions need further confirmation, however, the effectiveness of ECT or IRE, seem not to be impaired by the presence of a pacemaker or its leads in the vicinity of the treatment zone. Numerical modeling suggests that thermal damage due to heating of metallic casing of the pacemaker is unlikely.

## Methods

### Experimental evaluation of the effect of electroporation pulses on the pacemaker

The measurements were performed at room temperature (21 °C) in a glass container filled with either physiological saline (0.9% NaCl solution) or the saline diluted with distilled water at 1:4 ratio, thus resulting in two media with conductivities 1.57 and 0.34 S/m, respectively (the lower value mimicked the conductivities encountered in tissue during ECT of liver metastases). The conductivity was measured at 21 °C with SevenCompact S230 conductometer (Mettler Toledo, Columbus OH, USA). The experimental setup is presented in Fig. 4 [38].

In the first stage of the study, the bipolar ventricular lead (type CapSure Z Novus 5054) was not connected to the pacemaker; the connector (not submerged) allowed us to measure the maximum possible voltage or current (with the contacts open or shorted, respectively) between the pacemaker electrodes due to application of electroporation pulses. In the second stage, the ventricular lead was connected to an Adapta pacemaker (ADDR01 model, Medtronic, Minneapolis, USA) which was programmed into asynchronous D00 pacing mode and submerged. The atrial bipolar lead was also connected. Atrial pacing pulses were converted into adjustably delayed TTL pulses (0 V and 5 V output values) to trigger the generation of electroporation pulses. Thus, we were able to observe the effects on the pacemaker function for electroporation pulses delivered at different times with respect to the charge-balanced ventricular pacing pulse. The pacemaker is assumed to be in its most vulnerable state during generation of the pacing pulse due to relatively low internal impedance that could allow harmful currents flowing into the device due to electroporation interference.

Electroporation pulses were generated by Cliniporator Vitae device (IGEA, Carpi, MO, Italy) and delivered via two needle electrodes for clinical ECT (type VG-1230M20; conductive length 3 cm, diameter 1.2 mm) submerged in parallel into the medium (Fig. 4). The inter-electrode distance was fixed at 3 cm, the maximum distance limited by the hardware capacity and also recommended in the standard operating procedures for ECT [10]. Standard rectangular pulses (1000 and 3000 V amplitude, 100 µs duration) were generated individually or in sequences of four pulses (repetition rate 5 kHz, i.e., 100 µs on, 100 µs off). The voltages appearing between the electrodes of the ventricular lead were sensed at the ventricular electrodes in the medium. The measurement instrumentation included HDO6104A oscilloscope, two HVD3605 differential high-voltage and two CP031A high-current probes (Teledyne LeCroy, Chestnut Ridge, NY, USA) for monitoring of generated electroporation pulses and interferences on the ventricular lead.

### Numerical modeling

The impact of the presence of a metal-encased pacemaker on effectiveness and safety of electroporation-based therapies was further investigated by means of numerical computation. Two scenarios for treatment of a subcutaneous tumor were investigated: ECT and IRE. In both scenarios the influence of a metal-encased pacemaker was evaluated with the pacemaker in contact and without contact with one of the electrodes. A control scenario without the pacemaker was also evaluated. A previously designed numerical framework for planning of electroporation-based treatments was adapted for all computations [20, 28, 39].

All numerical computations were performed in COMSOL Multiphysics software (Comsol AB, Stockholm, Sweden), however the computations were set up and controlled in MATLAB (MathWorks, Natick, MA, USA) scripting environment through LiveLink. A simplified geometry including both the tumor and the pacemaker was used in this study. Placement of the pacemaker mimicked its position on the fascia of the pectoralis major muscle (Fig. 5). The tissue model consisted of three isotropic and homogeneous components: the spherical tumor (12 mm diameter), the fat tissue, and the underlying muscle tissue. The skin was not included in the model due to subcutaneous location of both the tumor and the pacemaker. The electrical and thermal properties of tissues and electrodes were taken from literature and databases and are listed in Table 1 along with the relevant references. The pacemaker model consisted of the titanium housing and the silicone-covered lead connectors. The built-in material properties from COMSOL were used (Titanium beta-21S and Silicon). An unstructured tetrahedral mesh was built in COMSOL and consisted of 27,643 elements for the ECT model and 21,623 elements for the IRE model. When compared to the finest possible mesh that was still manageable in the transient computation (197,119 elements for the ECT model and 138,480 elements for the IRE model), the use of a coarser mesh produced a < 1% error in calculated electric current and maximum temperature while greatly reducing the computation time.

**Table 1:** Electrical and thermal properties of modeled tissues taken from relevant literature (given in brackets)

	Fat	Tumor	Muscle
Initial electrical conductivity *σ*_0_ (S/m)	0.080 [40, 41]	0.200 [39, 42]	0.135 [35, 39]
Final electrical conductivity *σ*_end_ (S/m)	0.240 [35, 39]	0.600 [35, 39]	0.405 [35, 39]
Threshold for reversible EP (V/cm)	100 [35, 39]	400 [35, 39]	200 [35, 39]
Threshold for irreversible EP (V/cm)	900 [35, 39]	900 [35, 39]	900 [35, 39]
Thermal conductivity *k* (W/m K)	0.21 [41]	0.52 [20, 40]	0.49 [41]
Specific heat capacity *C*_*p*_ (J/ kg K)	2348 [41]	3540 [20]	3421 [41]
Density *ρ* (kg/m^3^)	911 [41]	1079 [41]	1090 [41]
Perfusion rate *ω* (1/s)	0.00043 [40]	0.01798 [40]	0.00069 [40]
Thermal coefficient of conductivity *α*_*T*_ (%/°C)	1.5 [20]	1.5 [20]	1.5 [20]

For the ECT model (Fig. 5a) a hexagonal-electrode configuration of seven electrodes was used with a standard ECT protocol: eight 100 μs pulses per each of 12 active electrode pairs delivered in two sequences of four pulses with reversed pulse polarities. The sequences were delivered at 1 Hz and the pulses within each sequence at 5 kHz repetition rate. The applied voltage was 730 V [6, 10].

For the IRE model (Fig. 5b) four needle electrodes were modeled, surrounding the tumor in a rectangular configuration. IRE delivery protocol from [20] was used in the simulation: 90 pulses of 90 μs duration per electrode pair with a 1500 V/cm voltage-to-distance ratio delivered at 1 Hz with a pause of 3 s after each set of 10 pulses. The pacemaker was positioned either 5 mm from the nearest electrode (Fig. 5a) or in direct contact with the nearest electrode (Fig. 5b).

Electric field distribution in tissue is determined through solving the stationary Laplace partial differential equation for electric potential. The outer boundaries of model domain are considered electrically insulated while the continuity equation is applied to the inner domain boundaries. Electroporation is implemented as a non-linear electric field dependent increase in tissue electrical conductivity [20]. Electric field distribution is calculated separately for each active electrode pair in the treatment. The computed electric field for the *n*-th electrode pair is compared to computed field from all previous pairs (1 to *n* − 1) and the maximum contributions from all pairs are combined into treatment equivalent field *E*_eq,*n*_ of *n*-th electrode pair as follows:

$${E}_{\mathrm{eq}, n}=\left\{\begin{array}{l}\mathrm{max}\left({E}_{\mathrm{eq},n-1},{E}_{n}\right); n>1\\ { E}_{n}; n=1\end{array}\right. ;\quad1\le n\le N,$$

where *N* is the total number of electrode pairs, *E*_eq,*n*_ is the treatment equivalent field after application of pulses to the *n-*th electrode pair, *E*_eq,*n−*1_ is the treatment equivalent field from electrode pairs 1 to *n* − 1 and *E*_*n*_ is the actual computed electric field produced by the *n*-th electrode pair. The final electric field distribution in tissue is represented by the equivalent electric field after application of pulses to all *N* electrode pairs (*E*_eq,*N*_). The percentage of tumor volume covered in target electric field strength, 400 V/cm for ECT and 650 V/cm for IRE ablation [39], was extracted from the final field distribution.

Computations of heat dissipation are performed separately with a transient model through solving the bioheat transfer equation [14, 28, 43]:

$$\rho {C}_{p}\frac{\partial T}{\partial t}+\nabla \bullet \left(-k\nabla T\right)=Q+\rho {C}_{p}\omega \left({T}_{\mathrm{blood}}-T\right)+{Q}_{\mathrm{met}},$$

$$Q=\sigma {\bullet E}^{2}.$$

**Fig. 6:**
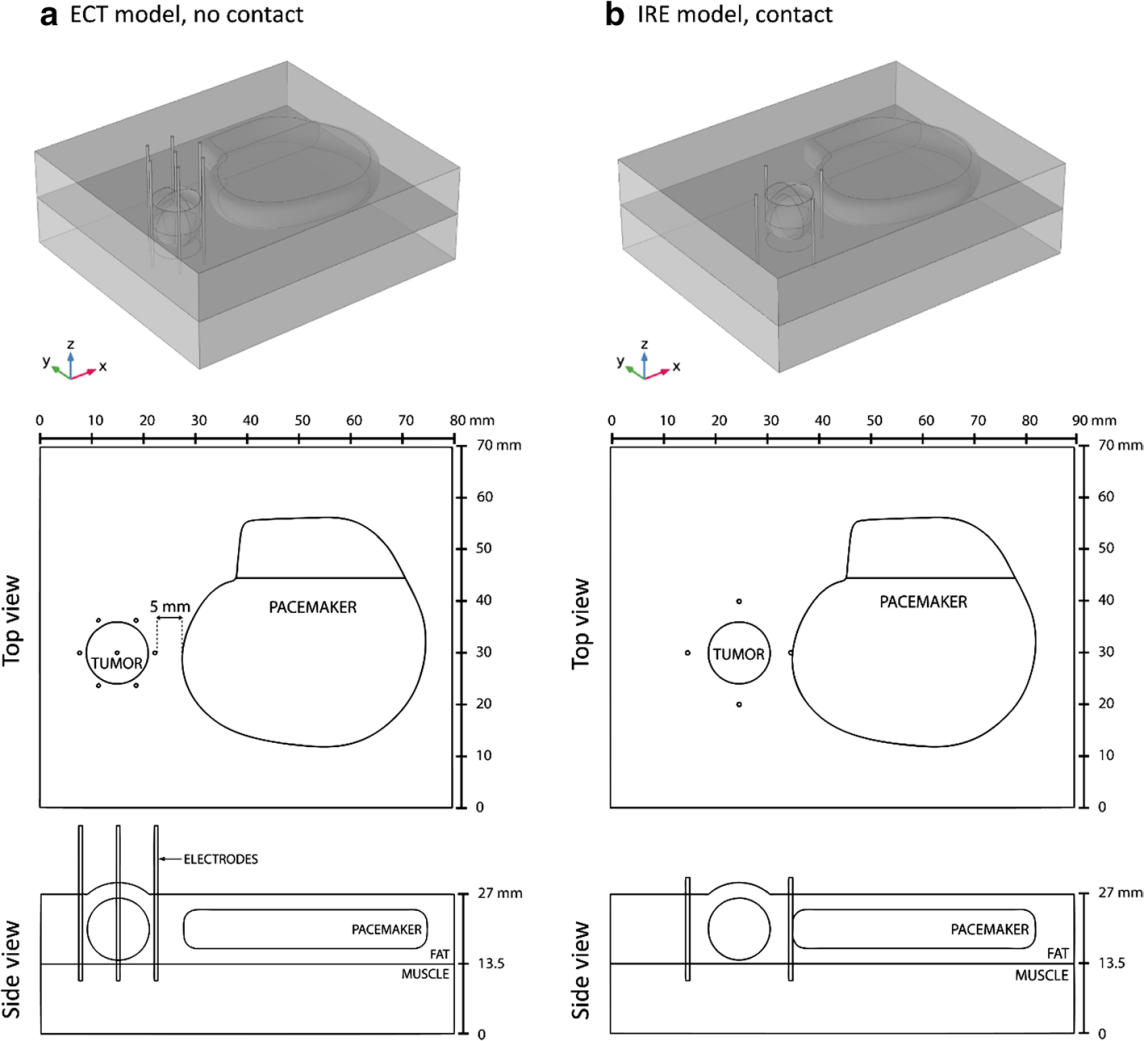
**a** 3D model for the simulation of ECT treatment of a spherical subcutaneous tumor. The distance from the pacemaker casing to the nearest electrode is 5 mm. **b** 3D model for the simulation of IRE ablation of a spherical subcutaneous tumor. Pacemaker is in direct contact with rightmost electrode. Only one scenario (contact or no contact) is shown for each treatment

Right side of the equation represents the heat sources in the model—the heat source *Q* approximated by a Joule heating term and source terms representing tissue perfusion and metabolism. Similarly to the computation of electric field distribution, the outer boundaries of the model domain are thermally insulated, in order to create the “worst case” conditions, while continuity condition is applied to the inner boundaries. All parameters descriptions and values are provided in Table 1. Maximum tissue temperature is calculated at the end of pulse delivery for each electrode pair (Fig. 6).

## Supplementary information

**Additional file 1.** Additional figures showing the interference of electroporation pulses delivered before, during and after the ventricular pacing pulse in the medium with a lower conductivity of 0.34 S/m. The figures show the effect of one pulse and a sequence of four pulses for pulse amplitude of 1000 V (**Figure S1**) and pulse amplitude 3000 V (**Figure S2**).

**Additional file 2.** Results of numerical computations. Additional tables containing results of numerical simulations for all six modeled treatment scenarios: delivered electric currents, maximum tissue temperatures, percentage of tumor volume covered in sufficiently high electric field (400 V/cm for ECT and 650 V/cm for IRE ablation) after delivery of pulses to each active electrode pair.

## Data Availability

The datasets generated and analyzed during the current study are not publicly available due to the still ongoing study, but are available from the corresponding author on reasonable request.

## Abbreviations

ECT: Electrochemotherapy
IRE: Irreversible electroporation
ECG: Electrocardiogram

## References

[1] Kotnik T, Rems L, Tarek M, Miklavčič D (2019). Membrane electroporation and electropermeabilization: mechanisms and models. Annu Rev Biophys.

[2] Yarmush ML, Golberg A, Serša G, Kotnik T, Miklavčič D (2014). Electroporation-based technologies for medicine: principles, applications, and challenges. Annu Rev Biomed Eng.

[3] Miklavcic D (2017). Handbook of electroporation.

[4] Marty M, Serša G, Garbay JR, Gehl J, Collins CG, Snoj M (2006). Electrochemotherapy—an easy, highly effective and safe treatment of cutaneous and subcutaneous metastases: results of ESOPE (European Standard Operating Procedures of Electrochemotherapy) study. Eur J Cancer Suppl.

[5] Mir LM (2006). Bases and rationale of the electrochemotherapy. Eur J Cancer Suppl.

[6] Miklavčič D, Mali B, Kos B, Heller R, Serša G (2014). Electrochemotherapy: from the drawing board into medical practice. Biomed Eng Online.

[7] Serša G, Miklavčič D, Čemažar M, Belehradek J, Jarm T, Mir LM (1997). Electrochemotherapy with CDDP on LPB sarcoma: comparison of the anti-tumor effectiveness in immunocompetent and immunodeficient mice. Bioelectrochem Bioenerg.

[8] Campana LG, Edhemovic I, Soden D, Perrone AM, Scarpa M, Campanacci L (2019). Electrochemotherapy—emerging applications technical advances, new indications, combined approaches, and multi-institutional collaboration. Eur J Surg Oncol.

[9] Clover AJP, Salwa SP, Bourke MG, McKiernan J, Forde PF, O’Sullivan ST (2020). Electrochemotherapy for the treatment of primary basal cell carcinoma; A randomised control trial comparing electrochemotherapy and surgery with five year follow up. Eur J Surg Oncol.

[10] Gehl J, Sersa G, Matthiessen LW, Muir T, Soden D, Occhini A (2018). Updated standard operating procedures for electrochemotherapy of cutaneous tumours and skin metastases. Acta Oncol.

[11] Geboers B, Scheffer HJ, Graybill PM, Ruarus AH, Nieuwenhuizen S, Puijk RS (2020). High-voltage electrical pulses in oncology: irreversible electroporation, electrochemotherapy, gene electrotransfer, electrofusion, and electroimmunotherapy. Radiology.

[12] Wagstaff PG, Buijs M, van den Bos W, de Bruin DM, Zondervan PJ, de la Rosette JJ (2016). Irreversible electroporation: state of the art. OncoTargets Ther.

[13] Aycock KN, Davalos RV (2019). Irreversible electroporation: background, theory, and review of recent developments in clinical oncology. Bioelectricity.

[14] Garcia PA, Davalos RV, Miklavcic D (2014). A numerical investigation of the electric and thermal cell kill distributions in electroporation-based therapies in tissue. PLoS ONE.

[15] Agnass P, van Veldhuisen E, van Gemert MJC, van der Geld CWM, van Lienden KP, van Gulik TM (2020). Mathematical modeling of the thermal effects of irreversible electroporation for in vitro, in vivo, and clinical use: a systematic review. Int J Hyperth.

[16] Scheffer HJ, Nielsen K, de Jong MC, van Tilborg AAJM, Vieveen JM, Bouwman ARA (2014). Irreversible electroporation for nonthermal tumor ablation in the clinical setting: a systematic review of safety and efficacy. J Vasc Interv Radiol JVIR.

[17] Verloh N, Jensch I, Lürken L, Haimerl M, Dollinger M, Renner P (2019). Similar complication rates for irreversible electroporation and thermal ablation in patients with hepatocellular tumors. Radiol Oncol.

[18] Ruarus AH, Vroomen LGPH, Geboers B, van Veldhuisen E, Puijk RS, Nieuwenhuizen S (2019). Percutaneous irreversible electroporation in locally advanced and recurrent pancreatic cancer (PANFIRE-2): a multicenter, prospective, single-arm phase II study. Radiology.

[19] Cohen EI, Field D, Lynskey GE, Kim AY (2018). Technology of irreversible electroporation and review of its clinical data on liver cancers. Expert Rev Med Devices.

[20] Kos B, Voigt P, Miklavcic D, Moche M (2015). Careful treatment planning enables safe ablation of liver tumors adjacent to major blood vessels by percutaneous irreversible electroporation (IRE). Radiol Oncol.

[21] Grošelj A, Kos B, Čemažar M, Urbančič J, Kragelj G, Bošnjak M (2015). Coupling treatment planning with navigation system: a new technological approach in treatment of head and neck tumors by electrochemotherapy. Biomed Eng Online.

[22] Miklavčič D, Davalos RV (2015). Electrochemotherapy (ECT) and irreversible electroporation (IRE)-advanced techniques for treating deep-seated tumors based on electroporation. Biomed Eng OnLine.

[23] Kalra N, Gupta P, Gorsi U, Bhujade H, Chaluvashetty SB, Duseja A (2019). Irreversible electroporation for unresectable hepatocellular carcinoma: initial experience. Cardiovasc Intervent Radiol.

[24] Frequently Asked Questions | IGEA. https://www.igea.it/en/oncology/information-clinicians/frequently-asked-questions. Accessed 20 Aug 2019.

[25] Risk Information. AngioDynamics. https://www.angiodynamics.com/about-us/risk-information/. Accessed 20 Aug 2019.

[26] Scheffer HJ, Vogel JA, van den Bos W, Neal RE, van Lienden KP, Besselink MGH (2016). The influence of a metal stent on the distribution of thermal energy during irreversible electroporation. PLoS ONE.

[27] Martin RCG, Durham AN, Besselink MG, Iannitti D, Weiss MJ, Wolfgang CL (2016). Irreversible electroporation in locally advanced pancreatic cancer: a call for standardization of energy delivery. J Surg Oncol.

[28] Cornelis FH, Cindrič H, Kos B, Fujimori M, Petre EN, Miklavčič D (2019). Peri-tumoral metallic implants reduce the efficacy of irreversible electroporation for the ablation of colorectal liver metastases. Cardiovasc Intervent Radiol.

[29] Dunki-Jacobs EM, Philips P, Martin RCG (2014). Evaluation of thermal injury to liver, pancreas and kidney during irreversible electroporation in an in vivo experimental model. Br J Surg.

[30] Faroja M, Ahmed M, Appelbaum L, Ben-David E, Moussa M, Sosna J (2013). Irreversible electroporation ablation: is all the damage nonthermal?. Radiology.

[31] Zmuc J, Gasljevic G, Sersa G, Edhemovic I, Boc N, Seliskar A (2019). Large liver blood vessels and bile ducts are not damaged by electrochemotherapy with bleomycin in pigs. Sci Rep.

[32] Korpas D (2013). Implantable cardiac devices technology.

[33] Bertacchini C, Margotti PM, Bergamini E, Lodi A, Ronchetti M, Cadossi R (2007). Design of an irreversible electroporation system for clinical use. Technol Cancer Res Treat.

[34] Garcia PA, Rossmeisl JH, Neal RE, Ellis TL, Davalos RV (2011). A parametric study delineating irreversible electroporation from thermal damage based on a minimally invasive intracranial procedure. Biomed Eng OnLine.

[35] Corovic S, Lackovic I, Sustaric P, Sustar T, Rodic T, Miklavcic D (2013). Modeling of electric field distribution in tissues during electroporation. Biomed Eng Online.

[36] Gallinato O, de Senneville BD, Seror O, Poignard C (2019). Numerical workflow of irreversible electroporation for deep-seated tumor. Phys Med Biol.

[37] López-Alonso B, Sarnago H, Burdío JM, Lucía O (2020). Electro-thermal modeling of irreversible electroporation and validation method of electric field distribution. Int J Appl Electromagn Mech.

[38] Jarm T, Krmac T, Miklavcic D, Magjarevic R. Cardiac Pacemaker exposed to electroporation pulses—an ex vivo study. In: Henriques J, Neves N, de Carvalho P, editors. XV mediterranean conference on medical and biological engineering and computing—MEDICON 2019 (IFMBE proceedings). Cham: Springer International Publishing; 2020. p. 439–46.

[39] Kos B, Županič A, Kotnik T, Snoj M, Serša G, Miklavčič D (2010). Robustness of treatment planning for electrochemotherapy of deep-seated tumors. J Membr Biol.

[40] Duck FA (2012). Physical properties of tissue: a comprehensive reference book.

[41] DATABASE » IT’IS Foundation. https://itis.swiss/virtual-population/tissue-properties/database/. Accessed 18 Feb 2020.

[42] Marčan M, Kos B, Miklavčič D (2015). Effect of blood vessel segmentation on the outcome of electroporation-based treatments of liver tumors. PLoS ONE.

[43] Lacković I, Magjarević R, Miklavčič D (2009). Three-dimensional finite-element analysis of joule heating in electrochemotherapy and in vivo gene electrotransfer. IEEE Trans Dielectr Electr Insul.

