# Supplementary material for: Investigation of safety for electrochemotherapy and irreversible electroporation ablation therapies in patients with cardiac pacemakers
Source: Biomed Eng Online. 2020 Nov 16;19:85. doi: 10.1186/s12938-020-00827-7 (PMC7667796; doi:10.1186/s12938-020-00827-7)
Supplement: Supplementary file 1 — Additional file 1. Additional figures showing the interference of electroporation pulses delivered before, during and after the ventricular pacing pulse in the medium with a lower conductivity of 0.34 S/m. The figures show the effect of one pulse and a sequence of four pulses for pulse amplitude of 1000 V (Figure S1) and pulse amplitude 3000 V (Figure S2). [file 12938_2020_827_MOESM1_ESM.docx]

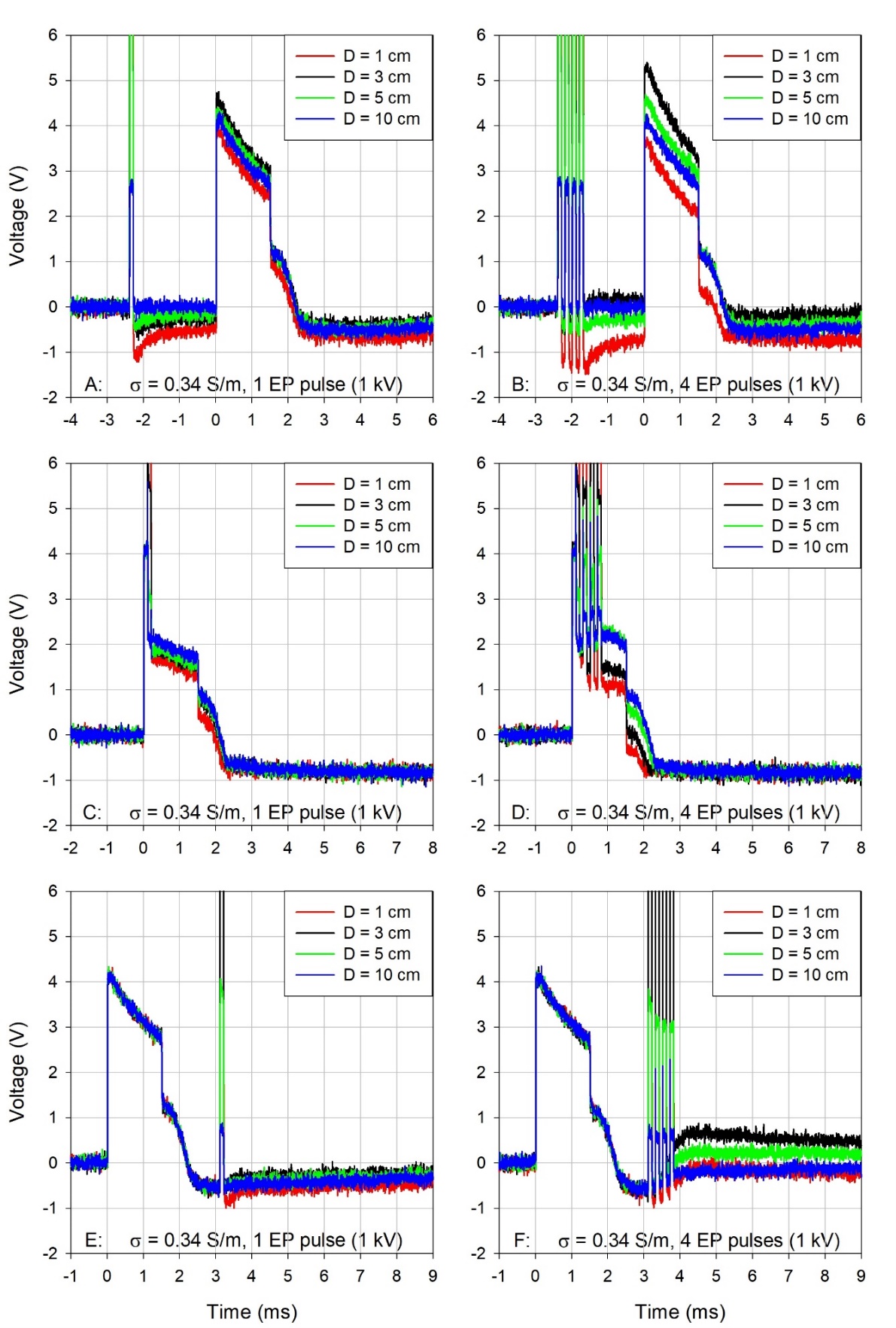


Figure S1: The interference of a single electroporation pulse (A, C, E) and a sequence of four electroporation pulses (B, D, F) with 1000 V amplitude, 100 microsecond duration, and 5 kHz repetition rate. Electroporation pulses were delivered before (A, B), during (C, D) or after (E, F) the ventricular pacing pulse in the medium with conductivity of 0.34 S/m


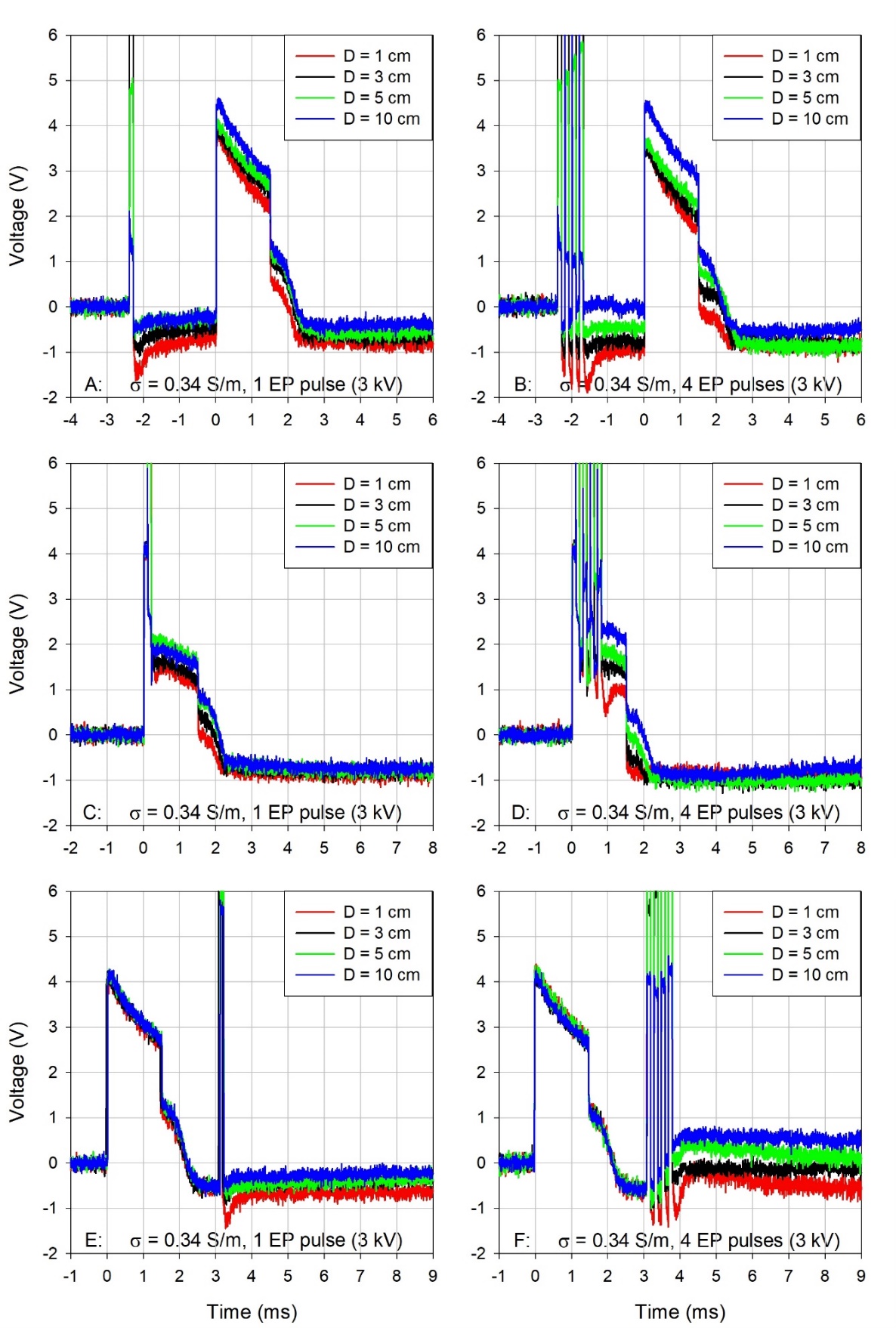


Figure S2: The interference of a single electroporation pulse (A, C, E) and a sequence of four electroporation pulses (B, D, F) with 3000 V amplitude, 100 microsecond duration, and 5 kHz repetition rate. Electroporation pulses were delivered before (A, B), during (C, D) or after (E, F) the ventricular pacing pulse in the medium with conductivity of 0.34 S/m


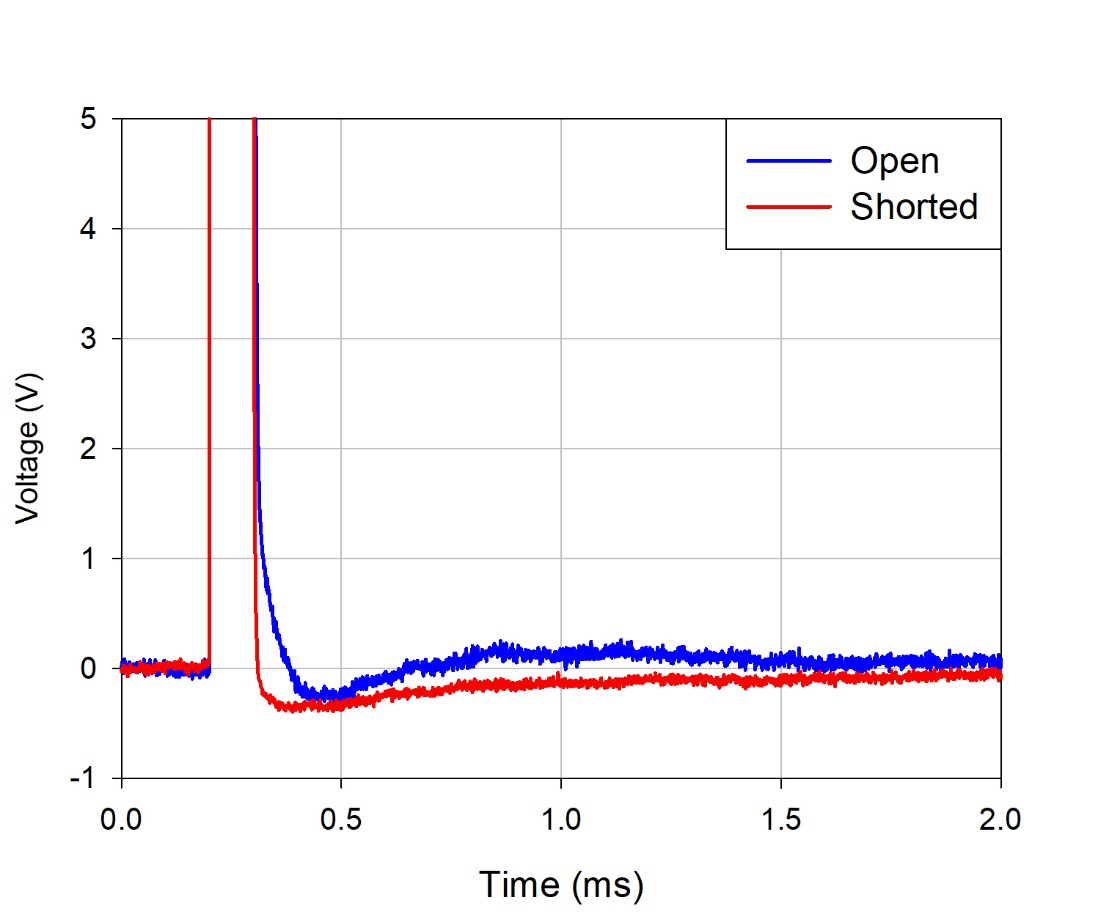


Figure S3: Transient decrease of the baseline voltage to negative values immediately following the electroporation pulse artefact as measured between the cathode and the anode of the pacemaker ventricular lead in the medium of 0.34 S/m conductivity for 1000 V pulse amplitude. Pacemaker was disconnected and the contacts of the ventricular lead, which would normally be plugged into the pacemaker, were either left open or shorted.
