# Supplementary material for: Investigation of safety for electrochemotherapy and irreversible electroporation ablation therapies in patients with cardiac pacemakers
Source: Biomed Eng Online. 2020 Nov 16;19:85. doi: 10.1186/s12938-020-00827-7 (PMC7667796; doi:10.1186/s12938-020-00827-7)
Supplement: Supplementary file 2 — Additional file 2. Results of numerical computations. Additional tables containing results of numerical simulations for all six modeled treatment scenarios: delivered electric currents, maximum tissue temperatures, percentage of tumor volume covered in sufficiently high electric field (400 V/cm for ECT and 650 V/cm for IRE ablation) after delivery of pulses to each active electrode pair. [file 12938_2020_827_MOESM2_ESM.docx]

Results of numerical computations

Control scenario: pacemaker is not present in the simulation

No contact scenario: pacemaker is positioned 5 mm from the surface of rightmost electrode

Contact scenario: pacemaker is in electric contact with the rightmost electrode

Table S1: Electrochemotherapy

| Electrode pair | Electric current [A] | | | Maximum tissue temperature [K] | | | Tumor volume coverage with 400 V/cm [%] | | |
| --- | --- | --- | --- | --- | --- | --- | --- | --- | --- |
|  | control | no contact | contact | control | no contact | contact | control | no contact | contact |
| 1 - 7 | 4.07 | 4.08 | 4.16 | 314.44 | 313.76 | 313.93 | 53.08 | 53.42 | 54.61 |
| 2*- 3 | 3.32 | 3.34 | 5.27 | 312.50 | 312.59 | 316.34 | 66.83 | 67.26 | 70.24 |
| 1 - 4 | 4.19 | 4.19 | 4.25 | 314.68 | 315.10 | 315.01 | 91.22 | 91.26 | 90.60 |
| 5 - 6 | 3.29 | 3.31 | 3.30 | 312.57 | 313.06 | 312.85 | 99.71 | 99.71 | 99.60 |
| 1 - 2* | 4.35 | 4.36 | 6.72 | 313.80 | 314.06 | 314.25 | 99.78 | 99.83 | 99.76 |
| 3 - 4 | 3.50 | 3.50 | 3.63 | 314.08 | 314.52 | 314.29 | 99.78 | 99.83 | 99.76 |
| 1 - 5 | 4.27 | 4.29 | 4.32 | 314.37 | 313.72 | 313.85 | 100.00 | 100.00 | 100.00 |
| 6 - 7 | 3.50 | 3.52 | 3.54 | 313.81 | 313.34 | 313.57 | 100.00 | 100.00 | 100.00 |
| 1 - 3 | 4.46 | 4.46 | 4.71 | 315.12 | 315.23 | 316.49 | 100.00 | 100.00 | 100.00 |
| 4 - 5 | 3.48 | 3.50 | 3.52 | 314.55 | 314.58 | 314.07 | 100.00 | 100.00 | 100.00 |
| 1 - 6 | 4.44 | 4.46 | 4.48 | 314.92 | 315.43 | 315.58 | 100.00 | 100.00 | 100.00 |
| 7 - 2* | 3.55 | 3.57 | 5.68 | 314.14 | 313.81 | 318.67 | 100.00 | 100.00 | 100.00 |
| 6 - 1 | 4.45 | 4.47 | 4.50 | 315.69 | 316.29 | 316.42 | 100.00 | 100.00 | 100.00 |
| 5 - 4 | 3.49 | 3.50 | 3.52 | 314.74 | 314.77 | 314.23 | 100.00 | 100.00 | 100.00 |
| 3 - 1 | 4.48 | 4.48 | 4.73 | 315.37 | 315.48 | 316.69 | 100.00 | 100.00 | 100.00 |
| 2*- 7 | 3.56 | 3.58 | 5.70 | 314.40 | 314.09 | 319.56 | 100.00 | 100.00 | 100.00 |
| 5 - 1 | 4.35 | 4.37 | 4.39 | 315.44 | 314.67 | 314.78 | 100.00 | 100.00 | 100.00 |
| 4 - 3 | 3.58 | 3.57 | 3.68 | 314.74 | 315.17 | 314.95 | 100.00 | 100.00 | 100.00 |
| 2*- 1 | 4.45 | 4.46 | 6.95 | 314.75 | 314.97 | 315.62 | 100.00 | 100.00 | 100.00 |
| 7 - 6 | 3.57 | 3.58 | 3.64 | 314.80 | 314.32 | 315.38 | 100.00 | 100.00 | 100.00 |
| 4 - 1 | 4.48 | 4.49 | 4.56 | 316.93 | 317.48 | 317.09 | 100.00 | 100.00 | 100.00 |
| 3 - 2* | 3.57 | 3.59 | 5.59 | 314.46 | 314.50 | 318.50 | 100.00 | 100.00 | 100.00 |
| 7 - 1 | 4.53 | 4.54 | 4.78 | 317.12 | 316.34 | 317.48 | 100.00 | 100.00 | 100.00 |
| 6 - 5 | 3.49 | 3.51 | 3.51 | 316.32 | 317.35 | 316.99 | 100.00 | 100.00 | 100.00 |

* contact electrode (where applicable)


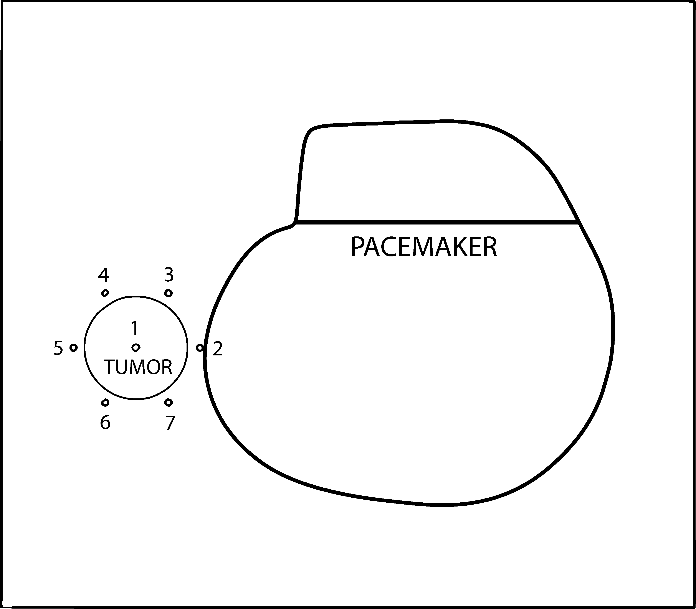


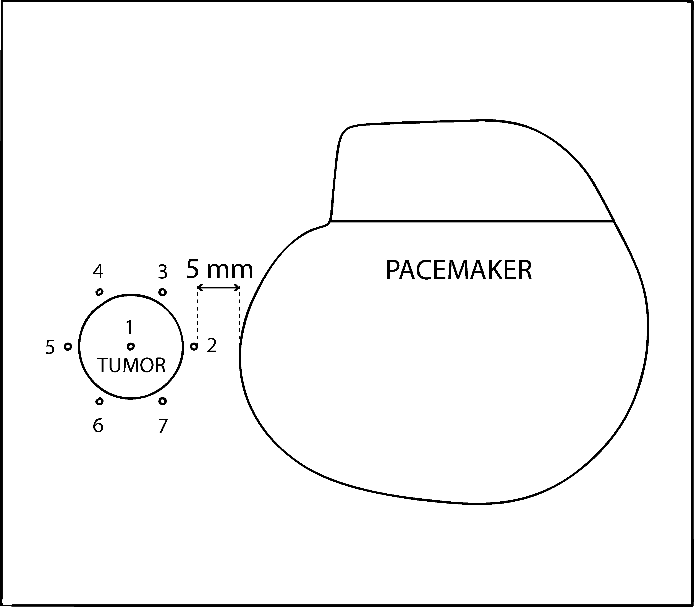
Figure S1: left – no contact scenario for ECT; right – contact scenario for ECT

Table S2: Irreversible electroporation ablation

| Electrode pair | Electric current [A] | | | Maximum tissue temperature [K] | | | Tumor volume coverage with 650 V/cm [%] | | |
| --- | --- | --- | --- | --- | --- | --- | --- | --- | --- |
|  | control | no contact | contact | control | no contact | contact | control | no contact | contact |
| 1*- 2 | 9.14 | 9.32 | 13.67 | 327.76 | 327.82 | 345.71 | 31.60 | 30.62 | 42.61 |
| 2 - 3 | 9.60 | 9.63 | 10.07 | 330.70 | 330.23 | 337.81 | 47.26 | 46.69 | 56.67 |
| 3 - 4 | 9.78 | 9.81 | 9.91 | 331.98 | 331.83 | 333.07 | 61.23 | 60.91 | 66.17 |
| 4 - 1* | 10.02 | 10.18 | 15.26 | 333.36 | 333.88 | 356.21 | 69.28 | 69.28 | 77.96 |
| 1*- 3 | 13.46 | 13.61 | 18.78 | 349.03 | 349.36 | 380.42 | 96.50 | 96.19 | 99.93 |
| 2 - 4 | 13.43 | 13.59 | 14.74 | 350.58 | 350.68 | 365.16 | 98.64 | 98.68 | 99.94 |

* contact electrode (where applicable)
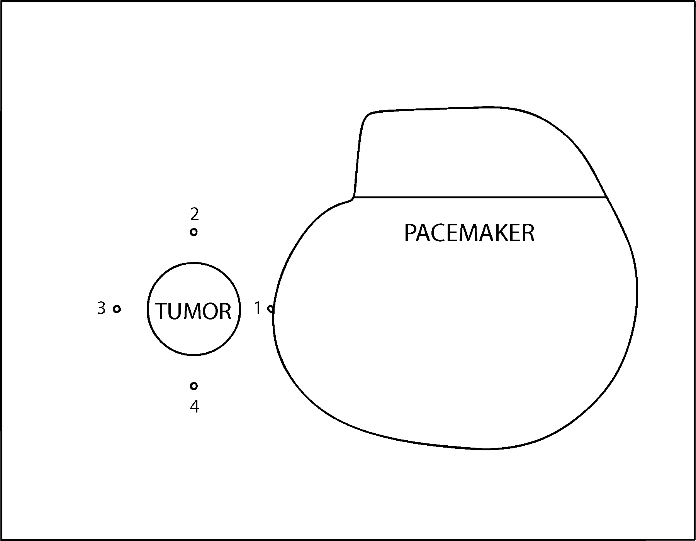

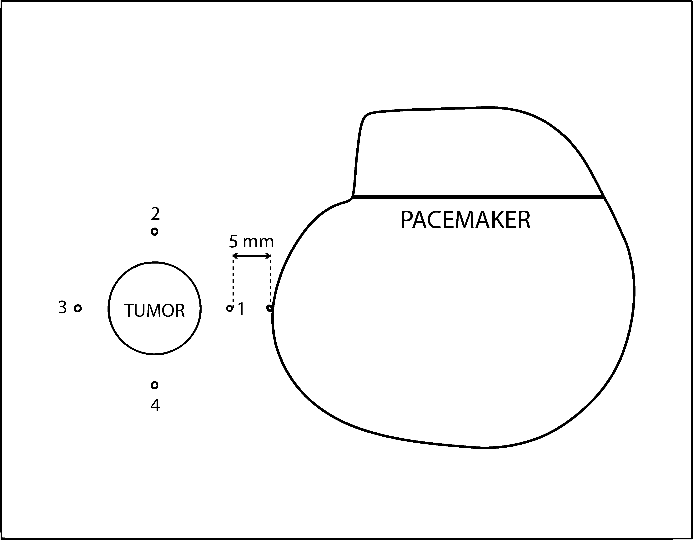


Figure S2: left – no contact scenario for IRE ablation; right – contact scenario for IRE ablation
